# Account of a Woman Who Had the Small Pox during Pregnancy, and Who Communicated the Same Disease to Her Fœtus

**Published:** 1781-09

**Authors:** 


II. *Account of a woman who had the ſmall pox during pregnancy, and who communicated the ſame diſeaſe to her fœtus. By* Robert Bland, *M. D. phyſician man-midwife to the Weſtminſter General Diſpenſary. Read* Sept. 10th, 1781.

SOME of the moſt reſpectable writers are found to differ in their opinion concerning the poſſibility of a pregnant woman's communicating to her fœtus the infection of the ſmall pox. A curious caſe related by the ingenious Mr. Hunter, in the 70th volume of the Philoſophical Tranſactions proves, that ſuch a communication may take place; but as a ſolitary fact, in a matter of this ſort, however ably ſupported, is ſeldom ſufficient to remove doubt, and as the number of caſes in this way can hardly ever be expected to be numerous, I ſhall make no apology for communicating to the Society an account of a ſimilar inſtance as it occurred lately to Mrs. Griffith, one of the Midwives to the Weſtminſter General

General Difpenfary, a perfon on whofe veracity I can depend. As the child was interred before I was informed of the fingularity of the cafe, I had no other means of fatisfying myfelf of the truth than by making a diligent inquiry concerning all the circumftances of it. This I have done, and as the account given me by Mrs. Gatton, the patient, confirm in every particular that of the midwife, I have no doubt of its having happened in the manner I fhall now relate.

In July laft Mary Gatton, of Prince's-ftreet, Weftminfter, was attacked with the fmall pox. She was then in the feventh month of her pregnancy. The difeafe proved to be of the confluent kind, and was attended with a confiderable fever. Six days after the turn of the pock, or about eighteen from the firft attack of the eruptive fever, fhe was taken in labour and delivered of a child, which feemed to have been dead five or fix days. Its body was covered with confluent fmall pox. The puftules were white and full of matter, and from their fize feemed to have nearly attained their maturity.

*St. Alban's-ftreet,*
Sept. 1, 1781.

SECTION

